# Exploring the structural and optical properties of lithium-chromium phosphate Li_3_Cr_2_(PO_3_)_4_

**DOI:** 10.1016/j.heliyon.2024.e36188

**Published:** 2024-08-14

**Authors:** Hajer Souissi, Souha Kammoun, Essebti Dhahri, E. López-Lago, B.F.O. Costa

**Affiliations:** a Applied Physics Laboratory, Luminescent Materials Physics Group, Faculty of Sciences in Sfax, Sfax University, BP 1171, 3000, Sfax, Tunisia; b Departamentode Física Aplicada, Facultade de Óptica e Optometría e Instituto de Materiais (iMATUS), UniversidadedeSantiago de Compostela, 15782, Galicia, Spain; c University of Coimbra, CFisUC, Physics Department, 3004-516, Coimbra, Portugal

**Keywords:** Li_3_Cr_2_(PO_4_)_3_ material, Neuhauser model, Crystal structure, Optical parameters, Crystal field theory

## Abstract

The Lithium-chromium phosphate Li_3_Cr_2_(PO_3_)_4_ sample was synthesized via the solid-state reaction method. The morphological integrity and chemical homogeneity were verified by energy dispersive X-ray analysis (EDX) and scanning electron microscopy (SEM). Infrared and Raman patterns were also analyzed. Optical absorption spectrum analysis, conducted within the range of 10000 cm^−1^ to 30000 cm^−1^ at room temperature, yielded some optical parameters (Eg, E_u_, δ, k, n). The Neuhauser model is used to interpret the interference dip which was on the absorption spectrum of Li_3_Cr_2_(PO_3_)_4_. The Fourier transform of the autocorrelation function leads to the Zero Phonon Lines of the observed absorption energies. The electronic structure of Cr^3+^ (3d (Huang et al., 2009) 33) ions in Li3Cr2(PO3)4 was calculated using Racah method, which allowed for precise calculations of Racah and crystal-field parameters. The results showed good agreement between the theoretical and experimental energy levels.

## 1 Introduction

Lithium metal phosphate materials Li_3_M_2_(PO_4_)_3_ with M is a transition metal ion (V, Fe, Mn, Cr …) are the newest generation of active materials. Li_3_M_2_(PO_4_)_3_ are also an intriguing materials with potential applications in various fields, including energy storage and optoelectronics [[1], [2], [3]]. Beyond their use in energy storage, knowing how structural and optical characteristics interact within Li_3_M_2_(PO_4_)_3_ creates opportunities to improve their efficiency, safety, and performance. The presence of chromium (Cr) within the structure offers opportunities to enhance the optical properties, and consequently particularly interesting for optical applications [4,5]. In fact, chromium, can introduce unique optical characteristics to the material. One notable aspect is its ability to exhibit different oxidation states, leading to diverse optical properties [6,7]. In the context of Li_3_Cr_2_(PO_4_)_3_, chromium likely occupies octahedral coordination sites within the crystal lattice, influencing its electronic band structure and optical behavior [8,9].

Moreover, the octahedral environment surrounding chromium ions can induce interesting optical phenomena, such as crystal field effects and charge transfer transitions. These effects can impact the absorption and emission spectra of the material, making it potentially useful for applications such as phosphor materials, solid-state lighting, or optical sensors [[10], [11], [12], [13]]. However, it's important to note that chromium is typically present in smaller amounts compared to phosphorus in Li_3_Cr_2_(PO_4_)_3._ While this may limit the extent of its influence on the material's optical properties.

This paper explores the intriguing properties of Li_3_Cr_2_(PO_4_)_3_, with a particular focus on the investigation of their optical and structural properties. First, we will concentrate on the Li_3_Cr_2_(PO_4_)_3_ compound's preparation and structural analysis. Next, we intend to present some important optical parameters: the band gap energy (E_g_), the Urbach energy (E_u_), the skin depth (δ), the extinction coefficient (k) and the refractive index (n). Furthermore, we analyze the feature observed in the absorption band of Cr^3+^ ions using the Neuhauser model. The objective of this study is to identify the cause of interference dips. Finally, a crystal field theory study was conducted for the Cr^3+^(3d [3]) in an O_h_ site symmetry using Racah tensor algebraic methods. It's notable that a few investigations have used crystal field theory to analyze the compound's absorption spectrum.

## 2 Experimental details

The Li_3_Cr_2_(PO_4_)_3_ compound has been prepared by the conventional solid-state reaction. The starting reagents Li_2_CO_3_, Cr_2_O_3_, and NH_4_H_2_PO_4_ were calculated in the desired proportion according to the following reaction:

3Li_2_CO_3_ + 2Cr_2_O_3_ + 6(NH_4_)_2_HPO_4_ → 2Li_3_Cr_2_(PO_4_)_3_ +3CO_2_ +12NH_3_+ 9H_2_O

The precursors are thoroughly mixed in equimolar proportions and ground in an agate mortar for 2h. The powder obtained is heated in a crucible at a temperature of 573K for 8h with intermediate regrinding to exhaust NH_3_, H_2_O, and CO_2_. Secondly, the calcined powders were uniaxial pressed into a cylindrical pellet using a hydraulic press and then the pellets were sintered at an optimized temperature 1100 K for 6h to acquire close packed grains.

A range of techniques was employed for the structural and optical characterization of phosphor. These techniques are included, scanning electron microscopy (SEM) coupled with energy dispersive X-ray (EDX) analysis, RAMAN, FTIR reflectance and absorption spectroscopy. Using a TESCAN VEGA3 SBH scanning electron microscope (SEM) equipped with an energy dispersive microscopy (EDS detector Bruker XFlagh 410 M), the morphology of the powder and its chemical composition were investigated. Using an image processing tool (Image J, version 1.50), at least 60 randomly chased grains were measured in order to collect size and size-distribution statistics from SEM pictures. The Fourier Transform Infrared Spectroscopy (FTIR) spectrum has covered a spectral range between 400 and 4000 cm⁻^1^, with the sample in solid form. The number of scans performed was 32, and the resolution was set at 4 cms⁻^1^, using a DTGS (Deuterated Triglycine Sulfate) detector. For the Raman spectrum, the laser wavelength used was 532 nm, covering a spectral range of 0–3600 cm⁻^1^. The Raman analysis also involved a solid sample, with 100 scans at a resolution of 30 accumulations, 0.5 s, detected by a CCD (Charge-Coupled Device)- black illuminate. For the evaluation of optical properties, we employed both reflectance and absorption spectroscopy techniques using the PerkinElmer Lambda 365 UV/Vis Spectrophotometer. This instrument, covering a wide wavelength range from 190 nm to 1100 nm, offers high resolution and sensitivity, crucial for detecting subtle spectral features.

## 3 Results and discussion

### 3.1 Morphological studies

As it is mentioned previously, Li_3_Cr_2_(PO_4_)_3_'s surface morphology, size distribution, and elemental composition were all thoroughly examined using an Energy Dispersive X-ray Spectroscopy (EDX) in conjunction with a Scanning Electron Microscope (SEM). To determine the size, shape, and morphological characteristics of the particles in the sample, SEM observations were used. The (SEM) micrograph of our compound is shown in Fig. 1 a. A detailed histogram of grain size was generated to analyze the particle size distribution (Fig. 1 b), providing quantitative insights into the dimensions of the particles comprising the material. The histogram of Fig. 1b shows a particle size that ranges from 0.2 to 1.2 μm, with an average size of 0.574 μm signifying the formation of micrometric particles. The fact that the particle is so small suggests that the synthesis of microparticles was efficient [14].

**Fig. 1. a:**
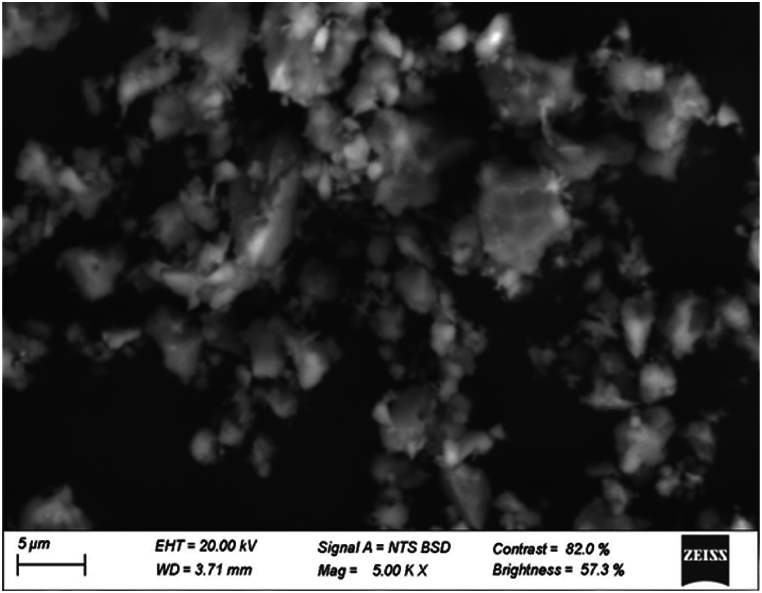
The SEM micrograph of Li_3_M_2_(PO_4_)_3_ around 5 μm.

**Fig. 1. b:**
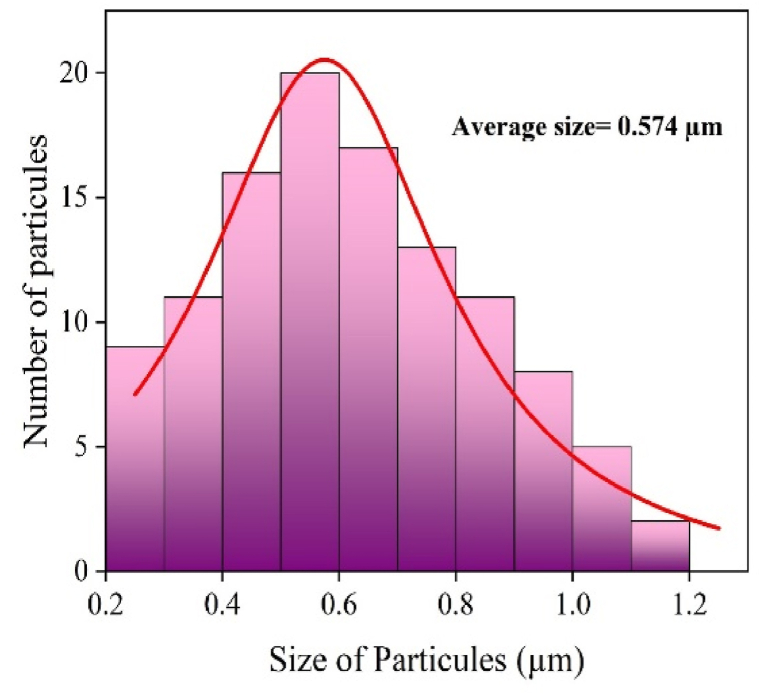
Histogram of grain size distribution of Li_3_Cr_2_(PO_4_)_3_.

**Fig. 2:**
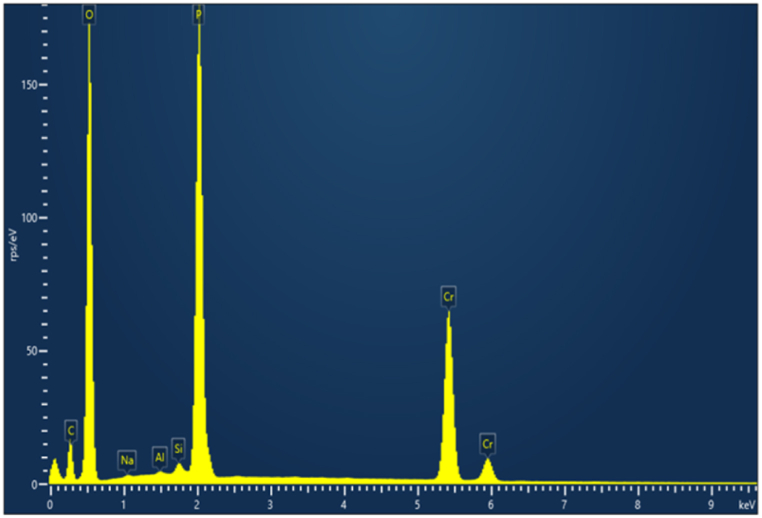
The quantitative analysis of the compositional elements presents in Li_3_Cr_2_(PO_4_)_3_ using EDX.

The EDX spectrum is displayed in Fig. 2. We notice that oxygen (O) is the predominant component, with phosphorus (P) and chromium (Cr) present in smaller quantities. Lithium (Li) is not detected in the EDX spectrum because it is a volatile element that tends to evaporate or sublimate when exposed to the electron beam used in EDX analysis. A non-negligible intensity peak labeled C is displayed in the spectrum. This peak corresponds to carbon emissions possibly coming from a carbon tape used during the measurement prosses. Fig. 2 shows also the presence of Na and Al, though in small quantities. These elements are considered impurities and may have originated due to contamination during sample preparation. The quantitative analysis of the compound's elemental composition as determined by EDX spectrum is summarized in Table 1.

**Table 1:** The elements present in Li_3_Cr_2_(PO_4_)_3_and their percentage of occupation.

Spectrum label	EDX Spectrum (%)
**C**	**16.25**
**P**	**18.51**
**O**	**42.89**
**Cr**	**22.34**
**Total**	**100.00**

### 3.2 Vibrational studies: FTIR and Raman

As described on the literature [[15], [16], [17]] Li_3_Cr_2_(PO_4_)_3_ crystallizes in a monoclinic structure, specifically in the space group P2_1_/n. The structure of our sample indeed consists of CrO_6_ octahedra that share corners with six PO_4_ tetrahedra. This creates a three-dimensional framework where the CrO_6_ octahedra are interconnected by the PO_4_ tetrahedra, contributing to the stability of the structure. This structural arrangement is common in various phosphate compounds [[15], [16], [17]]. The FTIR pattern for the compound under investigation is displayed in Fig. 3a. The vibrational features in the lower wavenumber region (400 –1000 cm^−1^) are like that of PO_4_^3−^ in Ref. [18]. The PO_4_^3−^group has a singlet (A_1_) at a frequency ν_1_ = 967 cm^−1^; a doublet (E) at ν_2_ = 447 cm^−1^ and two triply degenerate (T_2_) modes, ν_3_ at 1029 cm^−1^ and ν_4_ at 574 cm^−1^. The ν_1_ and ν_3_ modes involve the symmetric and anti-symmetric stretching vibrations of the P–O bonds, whereas ν_2_ and ν_4_ involve mainly O–P–O symmetric and antisymmetric bending mode [[19], [20], [21]]. The band at 1166 cm^−1^ region is owing to combined symmetric and antisymmetric mode [22]. Fig. 3b shows the Raman spectrum of our sample collected using a green laser line at 532 nm. The 300-376 cm^−1^ Raman components of Li_3_Cr_2_(PO_4_)_3_ are associated to Cr–O stretching modes of vibrations and are similar to those observed for Cr^3+^-O stretching modes in Na_3_Cr_2_(PO_4_)_3_ [23]. The band at 539 cm^−1^ in Raman spectrum corresponds to the attempts frequency of Lithium ions in the superionic [24]. Moreover, in the frequency region 900-1200 cm^−1^ (at 916–1086 – 1188 cm^−1^), The Raman bands are ascribed to the P–O stretching [18].

**Fig. 3.a:**
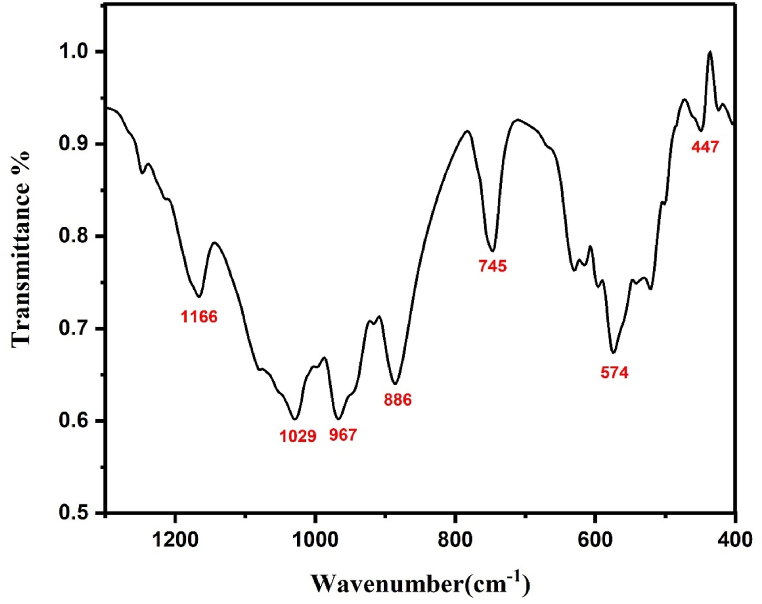
The FTIR spectrum of Li_3_Cr_2_(PO_4_)_3_ compound.

**Fig. 3.b:**
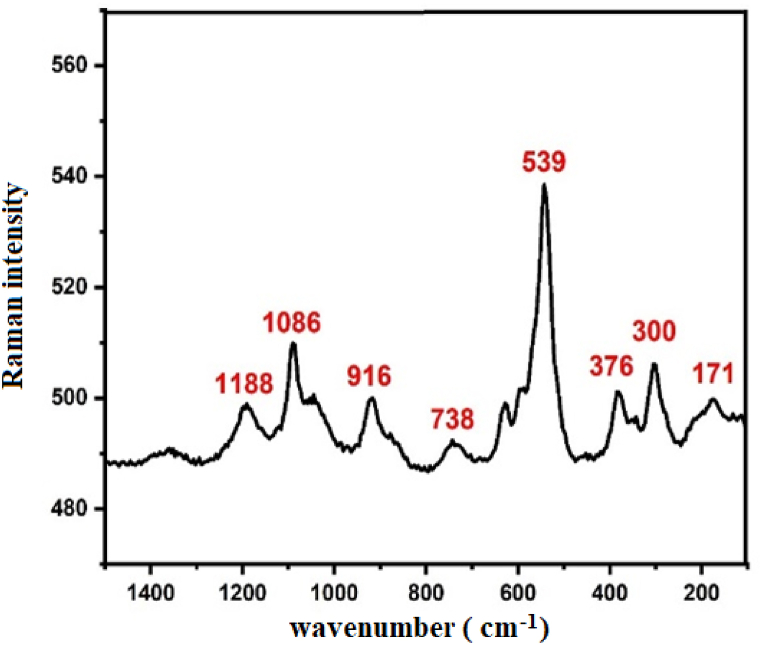
The Raman spectrum of Li_3_Cr_2_(PO_4_)_3_compound.

This part of our study is very interesting, especially in relation to the CrO_6_ octahedron. For complexes having an inversion symmetry center, vibronic origin is found to be a particularly useful method of increasing electron-dipole intensity during d-d transitions. Table 2 recapitulates the selection rules outlined for the O_h_ point group.

**Table 2:** Selection rules for Cr^3+^ d-d electronic transitions in O_h_ site symmetry for in Li_3_Cr_2_(PO_4_)_3_.

Electronic transition *O*_h_	Electric dipole allowed with Γ_u_ vibration $\left\langle \Gamma_{i}\left|T_{1u}\right|\Gamma_{f}\right\rangle \Gamma_{u}=A_{1g}$
^4^A_2g_ → ^4^T_2g_	a_1u_, t_1u_, t_2u_, e_u_
^4^A_2g_ → ^4^T_1g_	a_2u_, t_1u_, t_2u_, e_u_

Concerning the octahedron CrO_6_ octahedron, by eliminating the translation and rotation modes we have 15 internal vibration modes [4]:

(1) **Γ**_**vib**_ **=** **a**_**1g**_ **+** **e**_**g**_ **+** **t**_**2g**_**+ 2t**_**1u**_ **+** **t**_**2u**_

As shown in Equation (1), in our context, the t_1u_ mode is detected at 1166 cm^−1^ in Infrared spectroscopy, while Raman spectroscopy identifies activation of the a_1g_, e_g_, and t_2g_ modes [25]. Notably, the t_2u_ mode remains inactive in both spectroscopic techniques [25]. The observed d-d transitions are elucidated by vibronic coupling with the t_1u_ symmetry mode, which exhibits activity in Infrared spectroscopy. Notably, the vibronic frequency of the Raman-active a_1g_ mode is determined to be 376 cm^−1^, consistent with observations reported in previous studies.

### 3.3 Determination of some optical parameters

#### 3.3.1 Optical band gap energy Eg

**Fig. 4:**
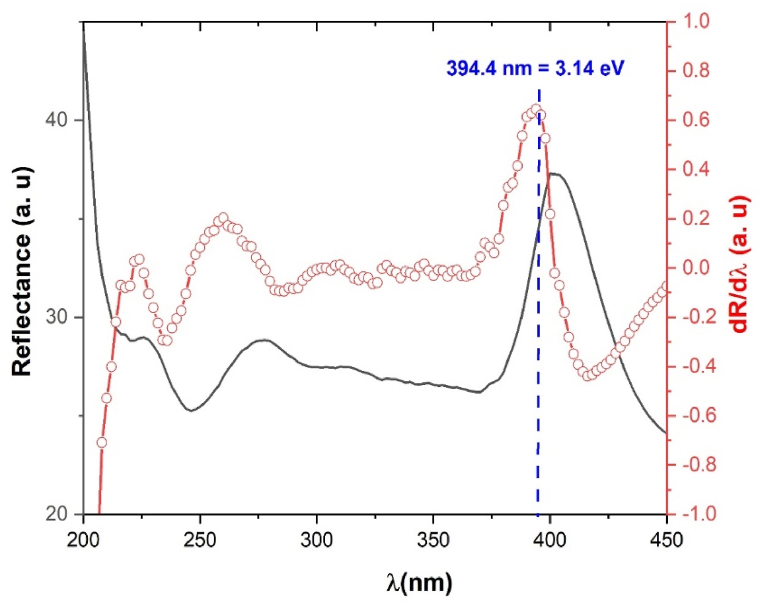
The spectrum of reflectance (dark solid line) and the evolution of dR/dλ(red circle) versus the wavelengthλ.

In this section we are going to determine the optical band gap Eg. We start our calculation by the Marotti method [26]. In this method the Eg parameter is determined directly from the maximum detected from the evolution of the spectrum dR/dλbeing R the reflectance and *λ* the wavelength. Fig. 4 shows the reflectance and the evolution dR/dλ spectra versus the wavelength *λ*. As it is clear we found the band gap E_g_ at 3.14 eV.

Another tool to determine the optical band gap is the Tauc method [27]. This method is a helpful technique for determining a material's optical bandgap (E_g_) from its absorption spectrum. This technique is also used especially for materials with a distinct absorption edge that matches the bandgap, such as semiconductors and insulators. The Tauc method uses the absorption spectrum which is represented in Fig. 5 a. The band gap energy (Eg) of Li_3_Cr_2_(PO_4_)_3_ was calculated using the widely recognized quadratic Tauc equation [27]:

(2) $$\alpha h\nu =A{\left(h\nu -E_{g}\right)}^{n}$$

Where α is the absorption coefficient, h is the Planck constant, ν is the photon's frequency, E_g_ is the band gap energy and A is a constant. The exponent value n depends on the nature of the band gap which is 1/2 for an allowed direct band gap and has the value 2 for an allowed indirect bandgap [28]. Fig. 5 b displays graphs of (αhν)^2^ and (αhν)^1/2^ plotted against photon energy (hν). Based on this analysis, we determined the optical energy band gaps for direct transition E_gd_ to be 2.95 eV and for indirect transition E_gi_ to be 2.46 eV.

**Fig. 5. a:**
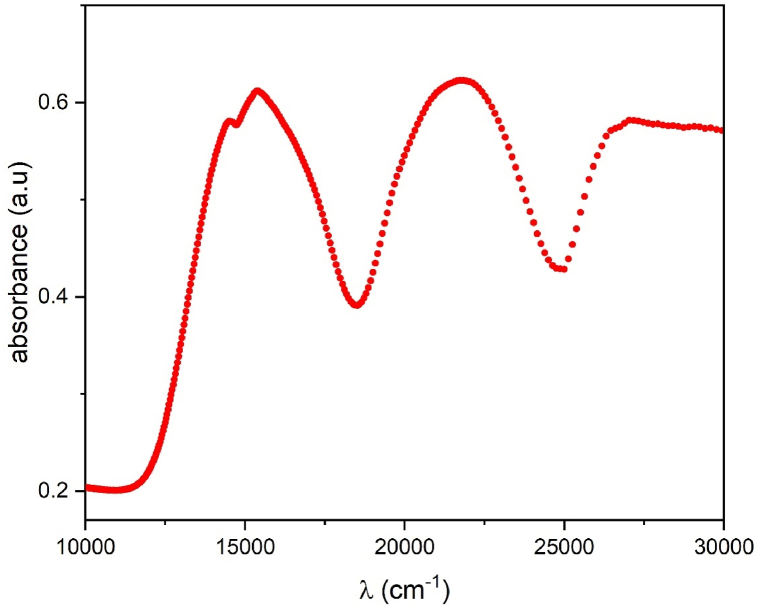
The absorption spectrum of Li_3_Cr_2_(PO_4_)_3_compound.

**Fig. 5.b:**
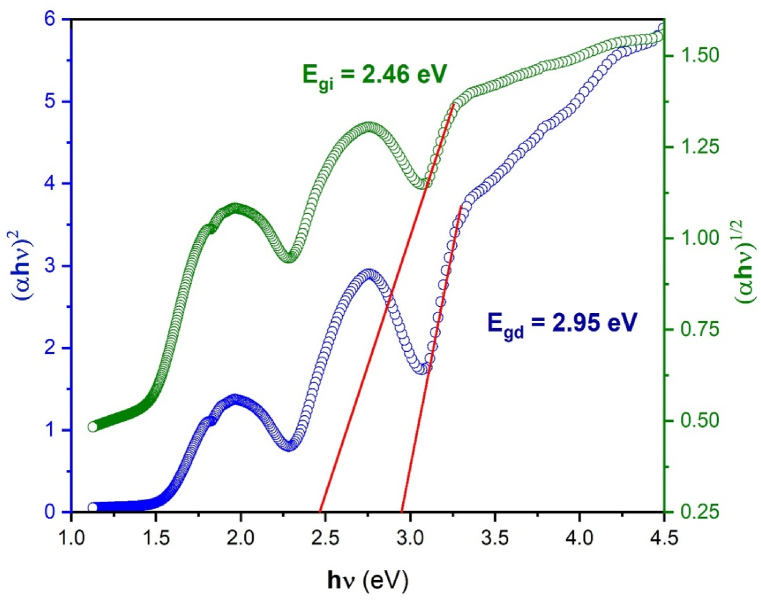
The evolution of (αhν)^1/2^and (αhν)^2^against (hν).

**Fig. 6:**
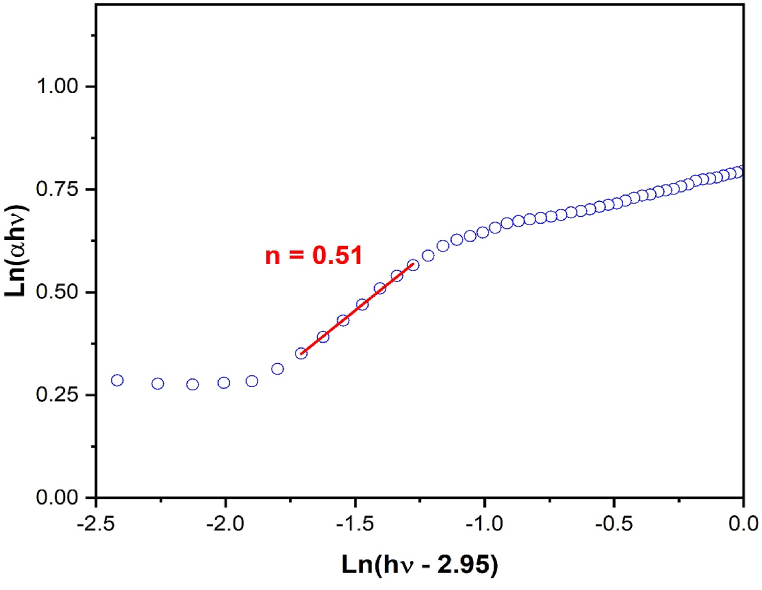
Evolution of Ln(αhν) against Ln(hν-2.95) for Li_3_Cr_2_(PO_4_)_3_.

We reorganize Equation (2) to confirm the type of optical band gap (whether direct or indirect), and we obtain the following relationship:

(3) $$\mathrm{Ln}\left(\alpha h\nu \right)=LnA+n\;\mathrm{Ln}\left(h\nu -E_{g}\right)$$

as shown in Equation (3), the plot of Ln(αhv) against Ln(hν-2.95) for Li_3_Cr_2_(PO_4_)_3_ is shown in Fig. 6. The power factor (n = 0.51), which is near to 0.5, is represented by the slope of the line in this plot, which depicts a linear relationship. According to this value, our sample coincides with wide optical band gap semiconductors that exhibit a direct band gap transition. The obtained value (2.95 eV) is in close agreement with the Marotti method result.

#### 3.3.2 Urbach energy Eu

The Urbach energy is a fundamental parameter in solid-state physics that characterizes the exponential tail of the absorption spectrum in a semiconductor or insulator. It is typically determined by the following relation [29]:

(4) $$\alpha \left(h\nu \right)=B\;\exp\left(\frac{h\nu -Eg}{E_{u}}\right)$$

**Fig. 7:**
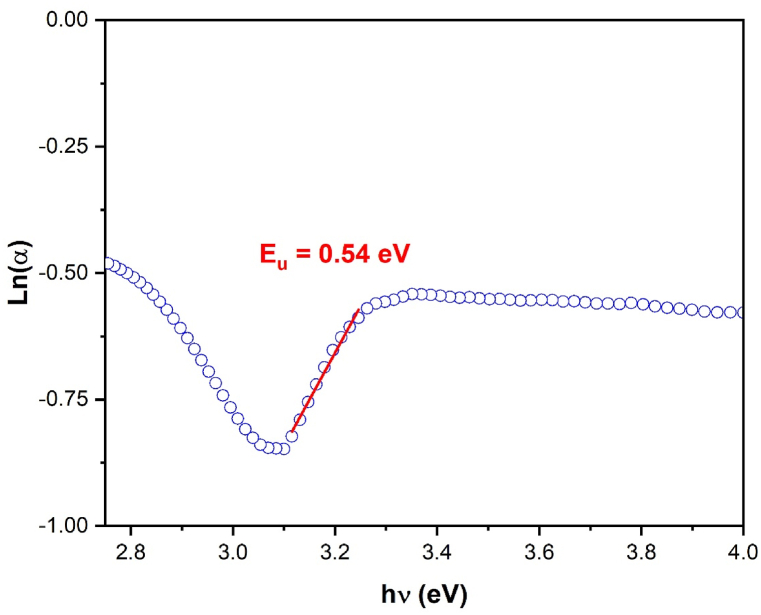
Curve of Ln(α) against hν to determine the Urbach energy for the Li_3_Cr_2_(PO_4_)_3_ compound.

The Urbach energy can be calculated based on Equation (4) by plotting (Ln(α)) against photon energy (hv) as illustrated in Fig. 7. The calculation of the Urbach energy is done by taking the reciprocal of the slope of the linear portion of this curve. Based on our analysis, we found that the band gap energy is around 18.3 % of the Urbach energy (Eu), which is 0.54 eV. This value indicates the density of localized states that exist within the compound, so verifying the existence of imperfections and structural disorder.

#### 3.3.3 The skin depth δ, the extinction coefficient k and the refractive index n of Li_3_Cr_2_(PO_4_)_3_

Penetration depth or alternatively optical depth or optical skin depth is a measure of how deep light can penetrate into a material before being significantly absorbed or attenuated. This parameter depends of the frequency of the light and it is from absorption coefficient (α) according to the formula [[30], [31], [32]]:

(5) $$\delta =\frac{1\;}{\alpha }$$

**Fig. 8:**
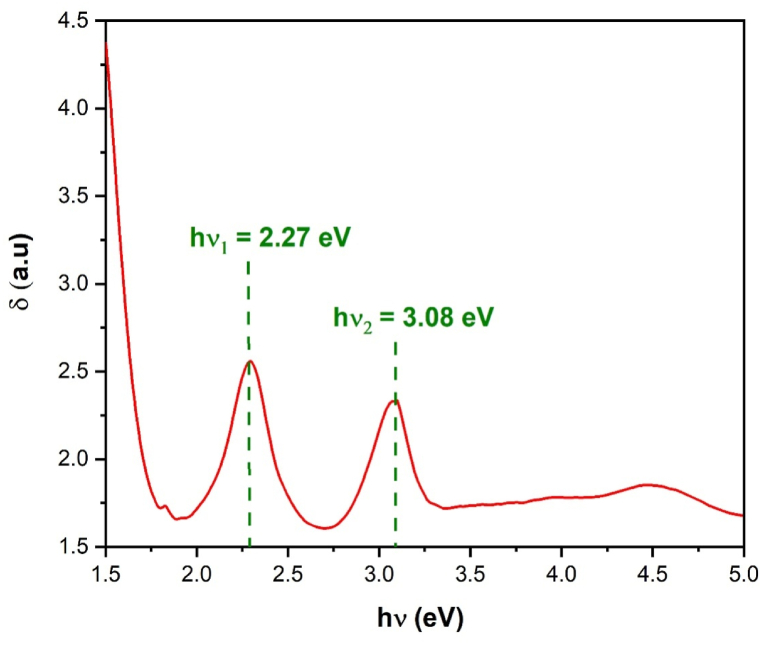
Skin depth (δ) against photon energy (hν) for Li_3_Cr_2_(PO_4_)_3_.

As shown in Equation (5), Fig. 8 shows the plot of skin depth δ versus (hν) for Li_3_Cr_2_(PO_4_)_3_. The spectrum exhibits the detection of two important penetration depths at specific energy levels within the visible region: hν_1_ = 2.27 eV and hν_2_ = 3.08 eV, signifies significant characteristics of materials' interactions with light in this spectral range. These particular penetration depths impact their optical behavior.

The absorption and scattering of light by a substance at a particular wavelength are quantitatively represented by the extinction coefficient k which is crucial in the design and characterization of optical materials and devices. The evolution of k parameter is determined by the following equation [26]:

(6) $$K\left(\lambda \right)=\frac{\alpha \lambda }{4\pi }$$

**Fig. 9:**
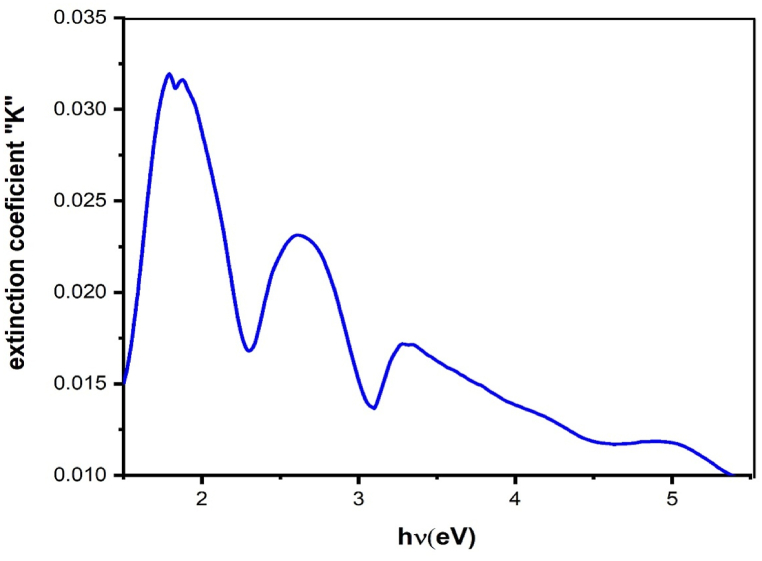
The evolution of the extinction coefficient (k) as a function of the wavelength hν.

According to Equation (6), Fig. 9 illustrates the evolution of k parameter versus hν. This figure demonstrates the presence of at almost three peaks in the visible region. These variations in the extinction coefficient are caused by variation in absorbance and the dark blue colour of the crystal.

Understanding the refractive index (*n*) of materials is important for designing and optimizing optical components and devices [[33], [34], [35]]. For reflectance and extinction, the coefficient n is defined by the following Equation (7) [36]:

(7) $$n=\frac{1+R}{1-R}+\sqrt{\frac{4R}{{\left(1-R\right)}^{2}}-k^{2}}$$

**Fig. 10:**
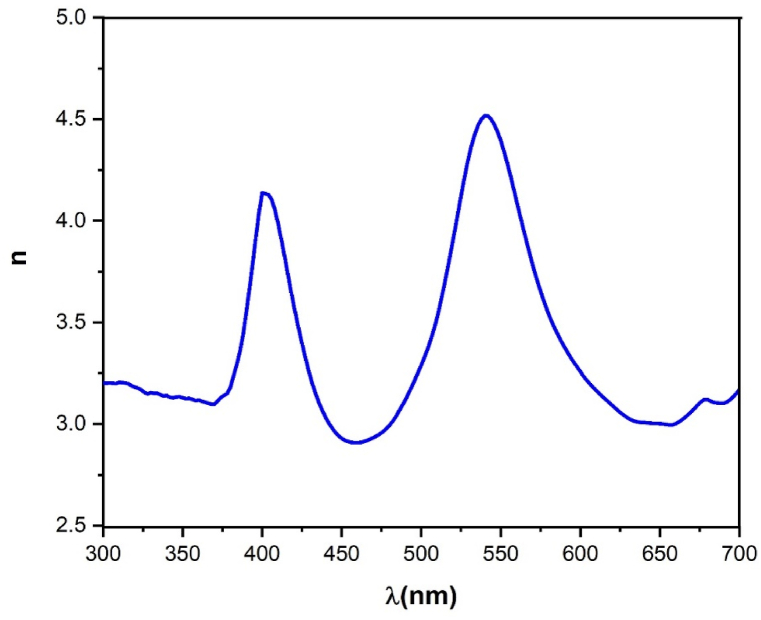
Refractive index (n) versus wavelength *λ* forLi_3_Cr_2_(PO_4_)_3_.

Fig. 10 shows the curve of refractive index against wavelength *λ*. This curve displays two important peaks at almost 402 nm (3.1 eV) and 541 nm (2.3 eV) which are very close to the maxima of the optical depth depicted in Fig. 8 and the minima of extinction displayed in Fig. 9.

## 4 The analysis of the absorption spectrum of Li_3_Cr_2_(PO_4_)_3_

### 4.1 Identification of interference dip in ^4^T_2g_(^4^F) exited state

**Fig. 11:**
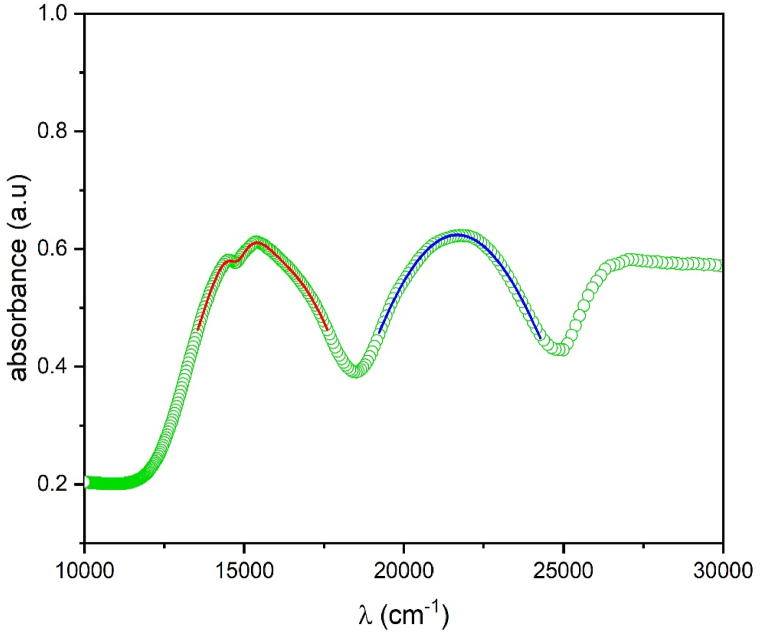
Absorption spectrum of Li_3_Cr_2_(PO_4_)_3_ phosphate and the results of the Neuhauser fit (Red colour), the Gaussian fit (blue colour).

**Fig. 12:**
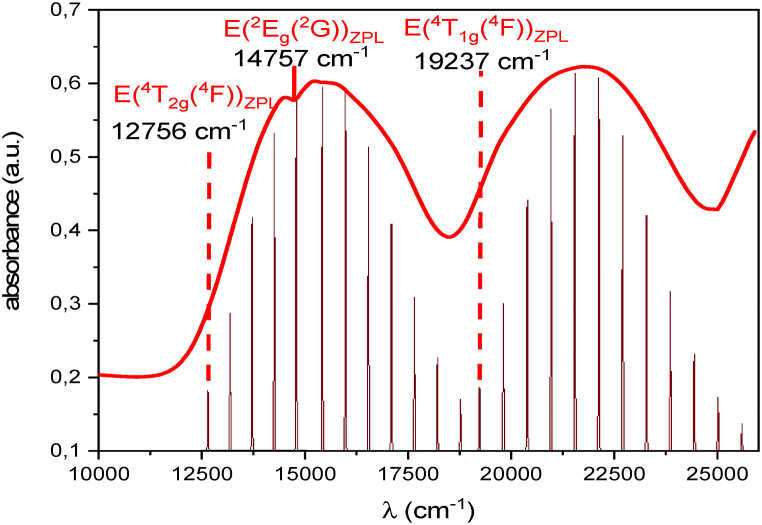
Modeling of the absorption spectrum for the of Li_3_Cr_2_(PO_4_)_3_ phosphate. The vertical arrows indicate the positions of the excited-state ZPL energies, E(^4^T_2g_(^4^F))_ZPL_, E(^2^E_g_(^2^G))_ZPL_ and E(^4^T_1g_(^4^F))_ZPL_.

In this part we will focus on the absorption spectrum of Fig. 11 recorded at ambient temperature. So, by examining the absorption features in this spectrum, we can elucidate the energy levels involved in the transitions and gain a deeper understanding of the electronic structure of the material. In this spectrum, two broad absorption bands are observed, corresponding to spin-allowed transitions from the ground state ^4^A_2g_(^4^F) to the excited states ^4^T_2g_(^4^F) and ^4^T_1g_(^4^F). Notably, an interference dip is observed within the broad band associated with the ^4^T_2g_(^4^F) transition. This interference dip is caused by the overlap of spin-forbidden transitions from the ground state ^4^A_2g_(^4^F) to the excited state ^2^Eg(^2^G). Our group is interested in understanding the likely features observed in absorption spectra [[37], [38], [39]] (see Fig. 12).

The interaction between states with distinct spin multiplicities is the source of the interference dip seen in this situation. In this work, we utilize the Neuhauser method for a main objective which is to accurately determine the levels of ^2^E_g_(^2^G) and the position of ^4^T_2g_(^4^F) state. All the details of the methodology outlined by Neuhauser et al. [40] and Bussière et al. [41] are presented in the following references [5,38,39].

In the case of Li_3_Cr_2_(PO_4_)_3_ the analytical expression describing the profile of the absorption spectrum is written as:

(8) $$\sigma \left(\omega \right)=-\frac{1}{\pi }\;Im\left[\frac{\beta }{1-\left(\gamma_{1}^{2}\alpha \right)\beta }\right]$$

As given by Equation (8), γ_1_ is the coupling constant of the forbidden state αwith the allowed one. ω is the frequency, β is the spectrum without coupling. The α and β equations are:

(9) $$\beta =\frac{1}{\omega -\Delta +i\sqrt{\omega_{0}\lambda_{e}}}$$

(10) $$\alpha =\frac{1}{\omega -\varepsilon_{F}+i\Gamma }$$

The width of individual vibronic lines is determined by Γ, a phenomenological damping factor, where Δ is the maximum of the absorption band without coupling and ε_F_ is the energies of the forbidden state. The width of the allowed band in Equation (9) is denoted by $\sqrt{\omega_{0}\lambda_{e}}$, where *λ*_e_ = Δ - ε_A_ and ω_0_ is the metal-ligand band's stretching frequency.

The absorption spectrum is fitted by:

(11) $$Re\left[\sigma \left(\omega \right)\right]=-\frac{A}{\pi }\frac{\frac{-\sqrt{\omega_{0}\lambda_{e}}-\;\frac{\Gamma \gamma^{2}}{{\left(\omega -\varepsilon_{F}\right)}^{2}+\Gamma^{2}}}{{\left(\omega -\Delta \right)}^{2}+\omega_{0}\lambda }}{{\left[1-\frac{\gamma^{2}\left(\left(\omega -\Delta \right)\left(\omega -\varepsilon_{F}\right)-\Gamma \sqrt{\omega_{0}\lambda_{e}}\right)}{\left({\left(\omega -\varepsilon_{F}\right)}^{2}+\Gamma^{2}\right)\left({\left(\omega -\Delta \right)}^{2}+\omega_{0}\lambda_{e}\right)}\;\right]}^{2}+{\left[\frac{\gamma^{2}\left(\left(\omega -\varepsilon_{F}\right)\sqrt{\omega_{0}\lambda_{e}}+\left(\omega -\Delta \right)\Gamma \right)}{\left({\left(\omega -\varepsilon_{F}\right)}^{2}+\Gamma^{2}\right)\left({\left(\omega -\Delta \right)}^{2}+\omega_{0}\lambda_{e}\right)}\right]}^{2}}$$

Equation (10), which is specifically programmed in Origin software, was used in the study to examine a single interference dip that was seen on the ^4^T_2g_(^4^F) band, as depicted in Fig. 11. The location of the dip is determined by this equation by calculating the energies associated with the ^4^T_2g_(^4^F) and ^2^E_g_(^2^G) states. After the software's calculation of these energies, the coupling (γ) and damping (Γ) parameters are important for the fitting process. The optimal values for γ and Γ, with other fitting parameters, are found in Table 3 and lead to the best fit.

**Table 3:** Fitting parameters in cm^−1^ for the calculation of the absorption spectra of Cr^3+^ in Li_3_Cr_2_(PO_4_)_3_.

Transitions	Δ(ε_A_(^4^T_2g_(^4^F)))	$\sqrt{\omega_{0}\lambda_{e}}$	ε_F_(^2^E_g_(^2^G))	Г	γ
^4^A_2g_(^4^F) → ^4^T_2g_(^4^F) (Neuhauserfit Eq (11))	15367	3038	14757	388	258
^4^A_2g_(^4^F) → ^4^T_1g_(^4^F) (Gaussian fit)	21365	–	–	–	–

ω_o_ = 376 cm^−1^ (totally symmetric Cr–O stretching mode of CrO_6_^3−^).

By applying the analytical Equation (10), the analysis successfully reproduces the observed dip by showing a strong correspondence between the computed results and the absorption spectrum. It is noteworthy to emphasize that the energy corresponding to the doublet state (ε_F_ = ^2^E_g_(^2^G)) is located at the depth of the dip instead of at the maximum of the peak. The parameter Δ = E(^4^T_2g_(^4^F)) represents the energy at the maximum peak of the wider band, roughly at the halfway point. The depth of these dips depends on the parameter Γ. γ = 258 cm⁻^1^ is the coupling constant between ^2^E(^2^G) and ^4^T_2_(^4^F). Furthermore, γ and the spin-orbit (S–O) coupling constant are related as γ = $-\xi \sqrt{\frac{6}{5}}$ [39], which results in ξ = 235 cm⁻^1^. For the band ^4^T_1g_(^4^F), the maximum of the peak is obtained by Gaussian fit and is represented in Table 3.

The Neuhauser method primarily allows for a precise reading of the spin-forbidden transition ^4^A_2g_(^4^F)→^2^E_g_(^2^G) due to the overlap between the broad band ^4^T_2g_(^4^F) and the narrow peak ^2^E_g_(^2^G) wich lead to interference deep. It's important to note that for the broad bands ^4^T_2g_(^4^F) and ^4^T1g(^4^F), the Neuhauser method fits a Gaussian band, and the obtained energies corresponds to the maximum of these bands. Additionally, it is worth noting that when considering the zero-phonon line, the ^4^T_2g_(^4^F) level appears before the ^2^E_g_(^2^G) level, which is the opposite of what is obtained with the Neuhauser method. For this reason we determine the zero-phonon line ZPL energies (E(^4^T_2g_(^4^F))_ZPL_ and E(^4^T_1g_(^4^F))_ZPL_ energies) of the Cr^3+^ ions from the absorption spectrum (Fig. 11). The E(^2^E_g_(^2^G))_ZPL_ energy is already determined from the Neuhauser method (Table 3). For determining the ZPL energies, we model the absorption spectrum (Fig. 11) by using the autocorrelation function. This function ⟨φ|φ(t)⟩ illustrates the dynamic evolution of the wave function from its initial state to its final state within a potential energy well. The Fourier transform of the autocorrelation function, which is the overlap of φ(t) with φ(t = 0), results in the absorption spectrum [4]:

$$I_{abs}\left(\omega \right)=C\omega^{3}\int_{-\infty }^{+\infty }e^{i\omega t}\left\{\left\langle \left\langle \varphi |\varphi \left(t\right)\right\rangle \right\rangle e^{-\Gamma^{2}t^{2}+i\frac{E_{ZPL}}{\hbar }t}\right\}dt$$

The evaluation of the autocorrelation function is simplified by assuming (i) the potential energy wells are harmonic with identical force constants for the initial and final states, (ii) a constant transition dipole moment, and (iii) identical normal coordinates for both states. Under these assumptions, the autocorrelation function can be expressed analytically as follows [4]:

$$\left\langle \varphi |\varphi \left(t\right)\right\rangle =\exp\left(\sum_{j}[-\frac{{\Delta Q}_{j}^{2}}{2}\left(1-e^{{ik}_{j}t}\right)-\frac{{ik}_{j}t}{2}]\right)$$

E_ZPL_ is the energy at the origin of the broad band (Zero Phonon Line), the frequency of the absorption spectrum is ω, and the breadth of each line in the spectrum is determined by the phenomenological damping factor Γ. For all amounts, wavenumber units are used. The terms k_j_ (frequency of 376 cm^−1^ is used) and $\Delta Q_{j}$ represent the vibrational frequencies of each mode and the displacement along the normal coordination Q_j_ of the potential surfaces, respectively. The experimental absorption spectrum is fitted by adjusting the parameters E(^4^T_2g_(^4^F))_ZPL_ and E(^4^T_1g_(^4^F))_ZPL_, Γ, and ΔQ. Table 4 lists the values that were obtained.

**Table 4:** Parameters used to calculate the absorption spectrum shown in Fig. 11.

parameters	values
E_ZPL_(^4^T_2g_(^4^F)) (cm^−1^)	12756
E_ZPL_(^2^E_g_(^2^G)) (cm^−1^)	14757
Γ (cm^−1^)	18
k (cm^−1^), ΔQ (dim,less)	376, 1.86
E_ZPL_(^4^T_1g_(^4^F)) (cm^−1^)	19237
Γ (cm^−1^)	20
k (cm^−1^), ΔQ (dim,less)	376, 2.21

### 4.2 Crystal field study of the Cr^3+^ in Li_3_Cr_2_(PO_4_)_3_

Understanding the electronic structure and behavior of transition metal complexes, especially coordination compounds, is possible through the application of Crystal Field Theory (CFT) framework. These complexes have ligands, or atoms or molecules that coordinate with the metal, all around a central metal ion. According to CFT, there is only electrostatic interaction between the metal ion and ligands. Ligands, which are usually negatively charged, surround the metal ion with a crystal field that affects the distribution and energy levels of its d-electrons. The following general Hamiltonian (Equation (12)) is utilized to ascertain the energy states of Cr^3+^ in Li_3_Cr_2_(PO_4_)_3_ compound [[42], [43], [44]]:

(12) **H = H**_**0**_**+ H**_**ee**_**(B,C) + H**_**Trees**_**(α) + H**_**CF**_**(Dq) + H**_**SO**_**(ζ)**

• H_0_ the Hamiltonian configuration
• H_ee_ the electron-electron repulsion term given as function of the Racah parameters (B and C) [[45], [46], [47]].In our case Cr^3+^ (3d [3]),the Russell-Saunders terms, representing the ^2S+1^L states, comprise ^4^F,^4^P,^2^H, ^2^G, ^2^F, and ^2^P terms.
• The H_Trees_ Hamiltonian relies on the fine structure parameter α_Tree_ and incorporates the Trees correction [48,49].
• H_CF_ is the crystal field Hamiltonian. In fact, in the case of Li_3_Cr_2_(PO_4_)_3_, The Cr^3+^ (3 d^3^) occupy an octahedral site symmetry and is given by the following equation [50,51]:

(13) $$H_{\mathrm{CF}}=21Dq\left[C_{0}^{\left(4\right)}+\sqrt{\frac{5}{14}}\left(C_{0}^{\left(4\right)}+C_{-4}^{\left(4\right)}\right)\right]$$

The ligand field splitting in the crystal field Hamiltonian H_CF_ is denoted by the parameter Dq, which is frequently used to describe the structure of transition metal ions. The matrix elements of the $C_{q}^{k}$ operators are numerically calculated using the Racah tensor algebraic methods [52]. The crystal field Hamiltonian H_CF_ is developed as a function of the Racah tensor operators $C_{q}^{\left(k\right)}$ defined as follows:

$$C_{q}^{\left(k\right)}=\sqrt{\frac{4\pi }{\left(2l+1\right)}}Y_{k}^{q}$$

$Y_{k}^{q}$ are the spherical harmonics.

For transition ions of the first series with electronic configuration 3d^N^, the crystal-field is of the intermediate strength [5,8,9], the basis functions $\left\{|d^{N}LSM_{L}M_{S}\rangle \right\}$ in the LS-coupling scheme have been adopted for the theoretical calculation.

By using the Racah tensor algebraic methods, the matrix elements of C_q_^(k)^ Racah tensor operators are calculated numerically [27]:

(14) $$\left\langle LSM_{L}M_{S}|C_{q}^{\left(k\right)}|L^{\prime }SM_{L^{\prime }}M_{S}\right\rangle ={\left(-1\right)}^{L-M_{L}}\left(\begin{array}{ccc} L & k & L^{\prime }\\ -M_{L} & q & M_{L^{\prime }} \end{array}\right)\left\langle LS\left\|C^{k}\right\|L^{\prime }S\right\rangle$$

According to Equation (14), the 3j-symbols $\left(\begin{array}{ccc} L & k & L^{\prime }\\ -M_{L} & q & M_{L^{\prime }} \end{array}\right)$ carry all the dependence on the labels M_L_, q and M_L_’ while the reduced matrix elements $\left\langle LS\left\|C^{k}\right\|L^{\prime }S\right\rangle$ is independent of these labels, but depends on the normalization of the tensor operators.

H_SO_isthe spin–orbit coupling Hamiltonian and is function of the ζ parameter which is the strength of the interaction between the orbital motion of an electron and its spin. The α_Tree_ and ζ parameters are calculated from the equations [26]:

(15-a) α_Trees_ = N^4^α_0_ and *ζ* = N^2^*ζ*_*0*_

where the parameter N describes the average reduction factor due to covalency [26]is defined as follows:

(15-b) $$N^{2}=\frac{1}{2}\left(\sqrt{\frac{B}{B_{0}}}+\sqrt{\frac{C}{C_{0}}}\right)$$

The free ion Cr^3+^ parameters B_0,_ C_0_, α_0_ and ζ_0_are illustrated in Table 5 [8,9].

**Table 5:** Crystal field, Racah, trees, spin-orbit coupling and nephelauxetic effect parameters values for Cr^3+^ in Li_3_Cr_2_(PO_4_)_3_.

Dq (cm^−1^)	1275
B (cm^−1^)	730
C (cm^−1^)	3190
α (cm^−1^)	23.504
ζ (cm^−1^)	243.414
Dq/B	1.75
C/B	4.37
β_B_	0.795
β_C_	0.772
B_0_ (cm^−1^)[8–9]	918
C_0_(cm^−1^)[8–9]	4133
α_0_ [8–9]	30
ζ_0_ [8–9]	275

The theoretical computation of energy levels for Cr^3+^ ions in Li_3_Cr_2_(PO_4_)_3_ involves diagonalizing the Hamiltonian Equation (13), which consists of a (120x120) matrix representing free ion eigenstates within quartet (^4^F ground state and ^4^P excited state) and doublet excited terms (^2^H, ^2^G, ^2^F, and ^2^P). A specialized code developed by our team, based on Maple software, is employed for diagonalization. The computed energies align closely with Yeung et al.'s findings [53], validating our computational approach. These energies, expressed as analytical equations, depend on parameters B, C, Dq, α, and ζ. Dq is determined from quartet excited state absorption energy, while B and C are derived from observed quartet and doublet excited states, respectively. Parameters α and ζ are calculated from Equation (15-a), (15-b). Inputting these parameters into our software package allows simulation of Cr^3+^ ion electronic structure and energy levels in Li_3_Cr_2_(PO_4_)_3_. The resulting parameters, detailed in Table 5, enable deduction of theoretical energy levels for Cr^3+^(3 d^3^) in O_h_ site symmetry (Table 6). Our computational approach, simplified by the developed software package, shows remarkable consistency between predicted Stark energy levels and actual experimental results.

**Table 6:** Experimental and calculated energies (cm^−1^) of Cr^3+^ in Li_3_Cr_2_(PO_4_)_3_.

O_h_	*E*_*obs*_	*E*_cal_* [this work]	*E*^*a*^_cal_* [this work]
^4^A_2g_(^4^F)	0	0	0
^4^T_2g_(^4^F)	12756	12750	12656 (2)
			12706 (4)
			12772 (4)
			12809 (2)
^2^E_g_(^2^G)	14757	14755	14964 (4)
^2^T_1g_(^2^G)			15578 (2)
			15678 (2)
^4^T_1g_(^4^F)	19237	19239	19063 (2)
			19088 (4)
			19239 (4)
			19286 (2)
^2^T_2g_(^2^G)	–	21420	21520 (4)
			21669 (2)
^2^A_1g_(^2^G)	–	25240	25447 (2)
^2^T_1g_(^2^P)	–	27421	27665 (2)
			27718 (4)
^2^T_1g_(^2^H)	–	27992	28058 (2)
			28242 (4)
^2^E_g_(^2^H)	–	29738	29653 (4)
^4^T_1g_(^4^P)	–	29960	29818 (2)
			29836 (2)
			29837 (4)
			30082 (4)
^2^T_1g_(^2^H)	–	32633	32692 (2)
			32786 (4)
^2^T_2g_(^2^H)	–	38006	37970 (4)
			38004 (2)
^2^A_2g_(^2^F)	–	39840	39853 (2)
^2^T_2g_(Da2)	–	40360	40663 (2)
			40772 (4)
^2^T_2g_(^2^F)	–	42741	42708 (2)
			42920 (4)
^2^E_g_(Da2)	–	46052	46139 (4)
^2^T_1g_(^2^F)	–	48074	47988 (2)
			48099 (4)
^2^E_g_(Db2)	–	62933	62909 (4)
			63591 (4)
^2^T_2g_(Db2)	–	63730	63748 (2)

**Fig. 13:**
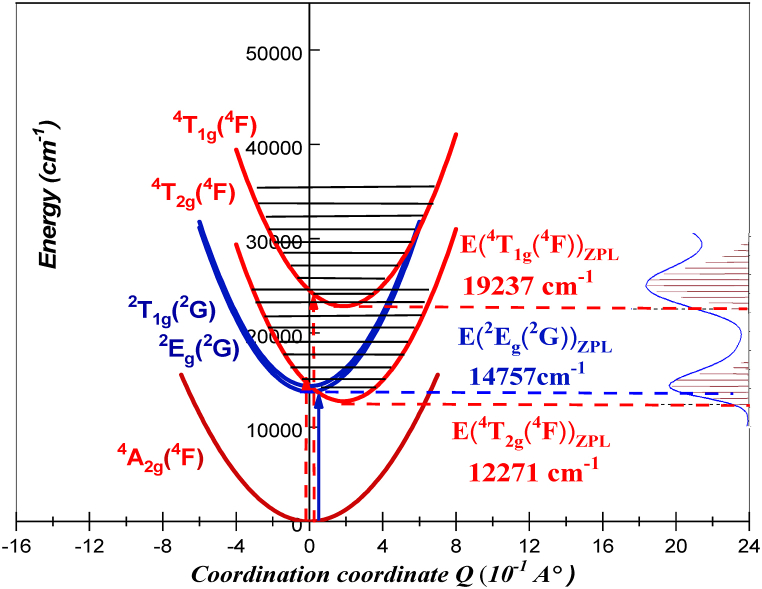
The diabatic potentials energy curves for ^2^E_g_(^2^G), ^4^T_2g_(^4^F) and ^4^T_1g_(^4^F).

An intriguing approach for comprehending the absorption spectrum is the coupled potential energy surface model. This approach allows for the examination of electronic transitions, particularly intra-configuration d-d excitations. A single normal coordinate, Q, is employed in the model of coupled potential energy surfaces. Notably, the doublet ^2^E_g_(^2^G) state and the ground ^4^A_2g_(^4^F) state share identical vibrational frequencies and positions of the potential energy minimum at Q = 0 Å [43]. However, a shift ΔQ causes the ^4^T_2g_(^4^F) state transitioning to longer metal-ligand bond distances. This change occurs because of the way certain molecular orbitals, specifically metal-ligand antibonding ones, are populated through a process called d-d excitation [43]. The potentials for the excited states ^2^E_g_(^2^G), ^4^T_2g_(^4^F) and ^4^T_1g_(^4^F) are as follows:

(16-a) V(E2g(G2))=12(kQ2)+EZPL(E2g(G2))

(16-b) V(T42g(F4))=12(k(Q–ΔQ1)2)+EZPL(T42g(F4))

(16-c) V(T41g(F4))=12(k(Q–ΔQ2)2)+EZPL(T41g(F4))

in the absorption spectrum, the breadth of the ^4^T_2g_(^4^F) and ^4^T_1g_(^4^F) bands corresponds to the magnitude of the displacements ΔQ. The E_ZPL_(^2^E_g_(^2^G)), E_ZPL_(^4^T_2g_(^4^F)) and E_ZPL_(^4^T_1g_(^4^F)) are the energies of the potential minimum for the ^2^E_g_(^2^G), ^4^T_2g_(^4^F) and ^4^T_1g_(^4^F) states, respectively. The autocorrelation function's modelling of the absorption spectra yielded the values of the quantities ΔQ, E_ZPL_(^2^E_g_(^2^G)), E_ZPL_(^4^T_2g_(^4^F)) and E_ZPL_(^4^T_1g_(^4^F)) (Table 4). In equation (15-a), (15-b), k is the frequency observed on the Raman spectrum of Li_3_Cr_2_(PO_4_)_3_ assigned to Cr–O symmetric stretching mode (a_1g_) denoted as ω_0_ (376 cm^-^1) in section 4-1. The potentials state surfaces, obtained from Equation (15-a), (15-b) and from the spectroscopic parameters shown in Table 4, are depicted in Fig. 13.

This diagram describes the electronic transitions at the zero-phonon line (ZPL) between the ground state ^4^A_2g_(^4^F) and the excited states ^2^E_g_(^2^G), ^4^T_2g_(^4^F) and ^4^T_1g_(^4^F).

Assuming that for Cr^3+^, the value of k is 0.21 [54], the bonding nature of Cr^3+^ is evaluated using the following expression [55]:

(17) $$h=\left[\frac{B_{0}-B}{B_{0}k}\right]$$

**Fig. 14:**
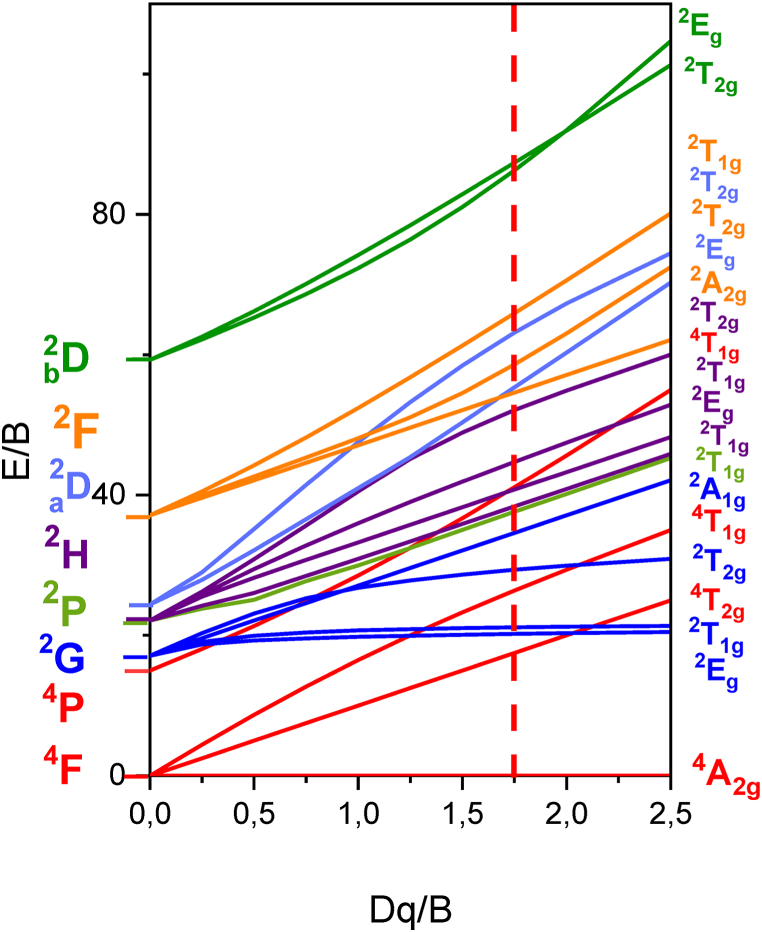
Tanabe-Sugano diagram for Cr^3+^ in an octahedral site symmetry. The vertical red line corresponds to the Dq/B = 1.75 obtained from our theoretical calculations.

Based on Equation (17), the calculation of h gives a value of **0.975**. This value indicates a d-electron delocalization, indicating predominant ionic bonding between Cr^3+^ and its ligands. Utilizing the Racah parameters B and C from Table 3 (with C/B = **4.37**), we construct the Tanabe-Sugano diagram for Cr^3+^ in an octahedral site symmetry (Fig. 14). This diagram illustrates the energy levels of Cr^3+^ ions concerning Dq/B as influenced by the local field strength. The specific case of Cr^3+^ in Li_3_Cr_2_(PO_4_)_3_ phosphate is represented by a vertical line corresponding to the Dq/B value obtained from our theoretical calculations.

**Fig. 15:**
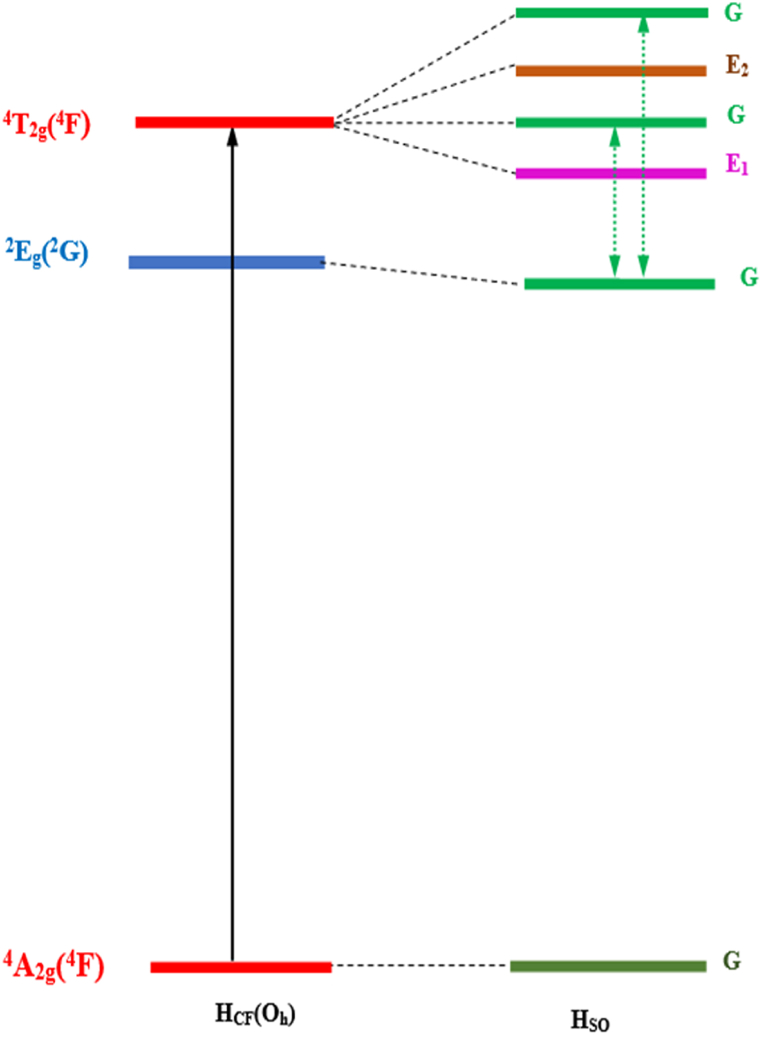
Splitting of Stark levels ^4^T_2g_ (^4^F) and ^2^E_g_ (^2^G) under spin-orbit coupling of Cr^3+^ in Li_3_Cr_2_(PO_4_)_3_. Spin-allowed transition is indicated by solid arrows. Under spin-orbit coupling, pairs of interacting levels are connected by dot-point arrows.

An additional method to elucidate the origin of interference dips involves examining the exchange of characteristics between the ^2^E_g_(^2^G) and ^4^T_2g_(^4^F) states, which exhibit proximal behavior. These two states may combine and exchange some of their respective characters. As illustrated in Fig. 15 through the process of spin-orbit coupling, the excited states ^2^E_g_ (^2^G) and ^4^T_2g_ (^4^F) can be divides into distinct E_1_, E_2_, and G states. Remarkably, these closely positioned states maintain an identical G character, facilitating a confluence of their attributes.

Consequently, the typically forbidden transition ^4^A_2g_(^4^F)→^2^E_g_(^2^G) assumes some features of the permitted transition ^4^A_2g_(^4^F)→ ^4^T_2g_(^4^F), resulting a gain in intensity which is lower than that of the allowed transition.

### 4.3 The diabatic/adiabatic approach for the interpretation of the forbidden transition

**Fig. 16:**
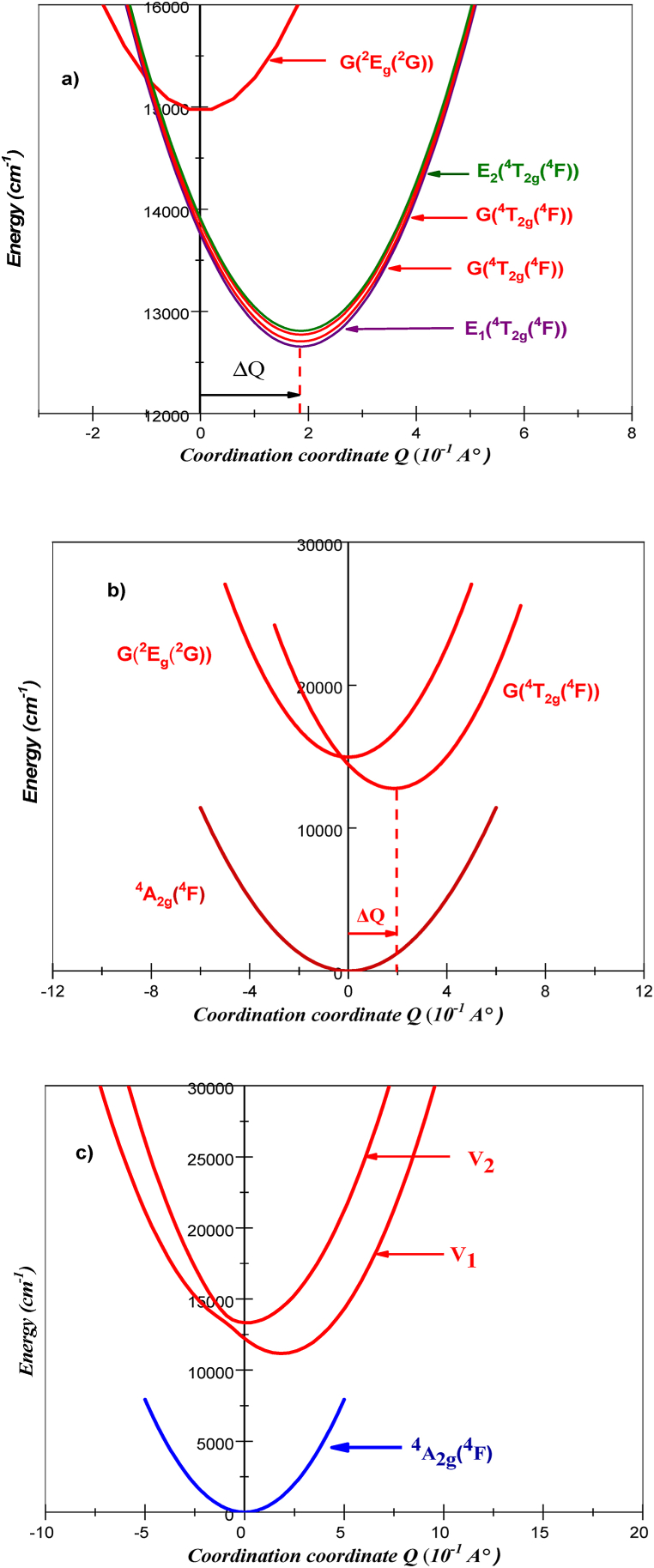
a) The uncoupled (diabatic) potentials for ^4^T_2g_(^4^F) and ^2^E_2g_(^2^G) subdivided into E_1_, E_2_, and G states b) we represent only the derivative states that have G irreducible representations. c) the coupled (adiabatic) excited state potential energy curves V_1_ and V_2_, (spin-orbit coupling between ^4^T_2g_(^4^F) and ^2^E_g_(^2^G) is taken into account).

As we have mentioned in previous, the existence of a dip in the ^4^T_2g_(^4^F) band, which is a forbidden transition. This feature can be caused by the interaction between states that are close to one another but have different spin multiplicities. As shown by Fano in atomic spectroscopy, these bands are not just additive but also closely connected, leading to interference phenomena [[37], [38], [39]]. To investigate the coupling between states of different spin multiplicities, we use again the model of coupled potential energy surfaces with a single normal coordinate Q. The potentials for the excited states ^2^E_g_(^2^G) and ^4^T_2g_(^4^F) in the absence of spin-orbit coupling are given by equation (15-a), (15-b). In this diabatic approach, the two potential wells of the excited states are considered independently of each other. So, Rapid nuclear motion prevents transitions between these wells, effectively sealing off any exchange of characteristics. The uncoupled (diabatic) potentials, resulting in the absence of spin-orbit coupling are illustrated in Fig. 16a. For the sake of simplification, only the derivative states that have G irreducible representations are displayed in Fig. 16b. The curves obtained from Equation (15a) and b cannot describe the spin forbidden transition ^4^A_2g_(^4^F) → ^2^E_g_(^2^G) detected in the absorption spectrum (Fig. 11).

Doublet excited state G(^2^E_g_) can interact with the G(^4^T_2g_) states. According to this analysis, the doublet states receive most of their intensities from the allowed quartet transitions and the spin forbidden transition ^4^T_2g_(^4^F)→^2^E_g_(^2^G) can arise from the interaction of the 3d [3] electrons of Cr^3+^ with the Li_3_Cr_2_(PO_4_)_3_ host matrix. The observed spin-forbidden transitions between the ^4^A_2g_(^4^F) ground state and the ^2^E_g_(^2^G) excited state are caused by the interaction between the same symmetry levels (G) derived from electronic states of different multiplicities [4]. Effectively, a constant with a magnitude proportional to the spin-orbit coupling *λ*($-3\lambda \sqrt{6/5}$) [55] are considered to mix the characters of the ^4^T_2g_(^4^F), and ^2^E_g_(^2^G) diabatic potentials. In this situation, the coupled potentials' matrix (adiabatic potentials) 2x2 is expressed in the following:

(18) V(Eg2,T42g)=(V(T2g4)−3λ6/5−3λ6/5V(Eg2))

The diagonals Terms in Equation (18) represent the equations of the diabatic potential energy wells and are therefore identical to those of Equation (16-c), (16-a), (16-b). The diabatic/adiabatic approach will considerably modify the appearance of the theoretical absorption spectrum and the appearance of observed forbidden transitions experimentally, such as the band corresponding to states ^4^T_2g_(^4^F) and ^2^E_g_ of Fig. 11, will appear in the calculation. The adiabatic potentials state surfaces, obtained from Equation (18) and from the spectroscopic parameters shown in Table 4, are depicted in Fig. 16c. The spin-forbidden ^4^A_2g_(^4^F) → ^2^E_g_(^2^G) transitions are caused by the adiabatic excited state potential energy curves V_1_ and V_2_, which are produced when the spin-orbit coupling between ^4^T_2g_(^4^F) and ^2^E_g_(^2^G) is taken into account.

## 5 Conclusion

This study focused on the synthesis and analysis of Li_3_Cr_2_(PO_4_)_3_ phosphate. Scanning electron microscopy (SEM) imaging exhibited particle dimensions spanning from 0.2 to 1.2 μm, with an average size of 0.574 μm, indicating the emergence of micrometer-scale particles. UV/Vis absorption spectra, assessed through Tauc's law and Urbach energy calculations, revealed direct transition properties, with increased disorder and defect density attributed to the presence of Cr^3+^ ions. Significantly, absorption spectra at room temperature displayed an interference dip within the extensive ^4^T_2g_(^4^F) band, ascribed to the overlapping of spin-forbidden transitions ^4^A_2g_(^4^F) → ^2^E_g_(^2^G). This phenomenon was clarified using the Neuhauser model, which relies on coupled potential energy surfaces. This model gives the ZPL energy of the ^2^E_g_(^2^G) level. The Fourier transform of the autocorrelation function leads to the Zero Phonon Lines of the observed absorption energies ^4^T_2g_(^4^F) and ^4^T_1g_(^4^F). From these ZPL energies, we determine a deeper understanding of the electronic configuration of Cr^3+^ (3 d^3^) ions within Li_3_Cr_2_(PO_4_)_3_ phosphate by using the tensoriel Racah theory. It aided in establishing Racah and crystal-field parameters, ensuring a good agreement between theoretical predictions and observed energy levels. The diabatic/adiabatic approach was an important model to demonstrate the origin of a dip in the ^4^T_2g_(^4^F). The obtained results hold significance for numerous optical applications.

## CRediT authorship contribution statement

**Hajer Souissi:** Writing – original draft. **Souha Kammoun:** Software. **Essebti Dhahri:** Visualization. **E. López-Lago:** Data curation. **B.F.O. Costa:** Supervision.

## Declaration of competing interest

This work includes an experimental study Lithium-chromium phosphate Li_3_Cr_2_(PO_3_)_4_ using the solid-state reaction method. The morphological integrity and chemical homogeneity were verified by energy dispersive X-ray analysis (EDX) and scanning electron microscopy (SEM). Infrared and Raman patterns were also analyzed. The optical properties have been study using the absorption characterization. An interference dip was found on the ^4^T_2g_(^4^F) band in the absorption spectrum of Li_3_Cr_2_(PO_3_)_4_, which was explained by spin-orbit coupling between the ^2^E_g_(^2^G) and ^4^T_2g_(^4^F) states. The electronic structure of Cr^3+^ (3d [3]) ions in Li_3_Cr_2_(PO_3_)_4_ was calculated using the Neuhauser model. The theoretical part is also carried out using software that we have developed ourselves in our laboratory and it is rarely that we find this type of calculation because this software is based on Racah method (the most powerful method that describe the electronic structure of transition ions) and it is not easy and requires specialists. The programming lasted about 6 years and each time we add more precision. This software uses Maple 20 as software is as well tested and gives the same result as another software using the Fortran software and we can prove that. We have published several articles and in well-indexed newspapers.
